# Formative research to understand food beliefs and practices relating to pregnancy on Kei Besar Island, Eastern Indonesia

**DOI:** 10.1186/s40795-024-00905-2

**Published:** 2024-07-11

**Authors:** Jessica Gloria Mogi, M Premikha, Ona Nabila, Adhi Sanjaya, Indira Prihartono, Joel Gittelsohn

**Affiliations:** 1 Master of Public Health, Johns Hopkins Bloomberg School of Public Health, Johns Hopkins University, Baltimore, MD USA; 2 Therapeutic Feeding Center, doctorSHARE/Yayasan Dokter Peduli, Southeast Maluku, Maluku, Indonesia; 3 Head Office, doctorSHARE/Yayasan Dokter Peduli, Central Jakarta Administrative City, Jakarta, Indonesia; 4 Department of International Health, Johns Hopkins Bloomberg School of Public Health, Johns Hopkins University, Baltimore, MD USA

**Keywords:** Food beliefs, Pregnant women, Qualitative study, Kei Besar, Maluku

## Abstract

**Background:**

Food-related beliefs and practices during pregnancy may contribute to the high prevalence of chronic energy deficiencies (CED) in Eastern Indonesia, particularly in Southeast Maluku regency, where 21.33% of pregnant women experience CED. Currently, little information on these issues is available. This study investigates food beliefs and practices related to pregnancy on Kei Besar Island in the Maluku province of Eastern Indonesia.

**Methods:**

A qualitative study was conducted utilizing in-depth interviews, free lists, and pile sort exercises. Data collection was conducted in January 2023 and involved married pregnant women aged 18 and above (*n* = 12), community health volunteers (*n* = 2), and traditional healers (*n* = 3) from 9 villages in Kei Besar District. All participants must be natives of Kei Besar Island, with community health volunteers and traditional healers being respective figures recommended by the local villagers.

**Results:**

The need to avoid or minimize consumption of certain foods during pregnancy, such as some kinds of fish, chili and spicy food, soda, pineapples, octopus, squid, and ice was reported by more than one-third of all participants. Consumption of prescribed foods, such as cassava leaves, papaya, coconut water, rice during early pregnancies, moringa leaves, bananas, and *katok* leaves was reported by five or more participants. These food proscriptions and prescriptions were due to concerns about the risks of miscarriage, adverse effects on the fetus and mother, and complications during labor. Participants also reported other practices, such as eating for two during early pregnancy and reducing food intake in late pregnancy. We found that food beliefs have shaped the dietary patterns of most participants. However, they still consumed food recommended by community health volunteers and midwives.

**Conclusions:**

Food beliefs are present and practiced in the Kei Besar community and may impact the nutritional status of women and their infants. Interventions should target training healthcare providers and community health volunteers to provide culturally appropriate health education that incorporates prescribed local ingredients and provides nutritionally adequate substitutes for the proscribed food items.

**Trial Registration:**

Not applicable.

**Supplementary Information:**

The online version contains supplementary material available at 10.1186/s40795-024-00905-2.

## Background

Maternal nutrition is an important factor in fetal development and growth, especially during the “first 1000 days of life” [1, 2], which starts from pre-pregnancy until a child is 24 months old. This period is considered to be a window of opportunity because it is a period of rapid development of the brain and other organs [2, 3]. Failure to meet nutritional needs during pregnancy can lead to maternal undernutrition or chronic energy deficiency (CED), defined as a lack of calorie and protein intake in women of reproductive age [4, 5].

CED is a persistent problem in low-and middle-income countries that places the mother at greater risk of complications during childbirth, such as premature birth and postpartum hemorrhage [5–7]. Chronic energy deficiency in mothers during pregnancy also contributes to low birth weight, placing babies born with low birth weight at a higher risk of stunting and even mortality if untreated with the appropriate intervention [7]. Being stunted affects their cognitive development, and subsequently, their performance in school and as adults [8]. Stunting, severe wasting, and intrauterine growth restriction accounted for 2.2 million deaths for children younger than 5 years [7]. Moreover, in 2016, child stunting contributed to 14,114,740 disability-adjusted life years (DALYs) [9].

Overall, the prevalence of CED among pregnant women in Indonesia decreased from 17.3% in 2018 to 8.7% in 2021, 5.8% lower than the 2021 Indonesian Ministry of Health target of 14.5% [10]. Despite this remarkable achievement, CED prevalence remains above 14.5% in some provinces in Eastern Indonesia, namely Maluku, East Nusa Tenggara, South Sulawesi, Southeast Sulawesi, Papua, and West Papua [10]. According to The Ministry of Health, poor nutrition education and local cultural practices that forbid pregnant women from consuming certain foods are potential contributors of CED in these provinces [10].

As far as the authors’ research, no study of these beliefs and practices has been conducted on Kei Besar Island, an island within the Maluku Province. Southeast Maluku, the regency that houses Kei Besar Island, is known for its protein-rich sea produce [11]. However, 21.3% of their pregnant women have CED and more than 28% of children under-two are stunted, making Southeast Maluku one of the regencies with the highest stunting rates in Maluku [12]. Studies conducted in other areas of Eastern Indonesia with high CED prevalence, such as Central Maluku, Boven Digoel, and Sumbawa [13–15], mentioned some foods believed to bring adverse effects to the pregnant women’s fetus. These food include certain seafood (ray fish, dried scaly fish, crabs, skipjacks, squids), starches (sago, cassava, sweet potato, fermented sticky rice), and vegetables and fruits (pumpkins, cabbages, pineapples, and bananas) [13–15]. Similar beliefs and practices may also exist in Kei Besar Island. Given the high prevalence of malnutrition on Kei Besar Island, there is a gap in understanding the beliefs and practices surrounding food consumption among the population, highlighting the need for studies to inform local authorities and stakeholders about the potential interventions to address this issue.

This study investigated the food beliefs and practices relating to pregnancy in Kei Besar Island to guide intervention development, and addressed the following questions:

- What are the beliefs of Kei Besar people regarding foods that *should not* be consumed during pregnancy (food proscriptions)?
- What are the beliefs of Kei Besar people regarding foods that *should* be consumed during pregnancy (food prescriptions)?
- How do these beliefs potentially impact actual dietary behavior during pregnancy?

## Methods

We conducted a qualitative study of food beliefs and practices during pregnancy on Kei Besar Island, using a combination of semi-structured interviews and cognitive interviewing methods with pregnant women, community health volunteers (*kader kesehatan*), and traditional healers (*mama biang*).

### Study setting descriptions

Kei Besar Island (Great Kei Island) is one of the main islands in Southeast Maluku Regency of Maluku province, the other being Kei Kecil (Lesser Kei Island) [16]. The island is divided into 5 main districts with a total area of 558.83 km^2^ and approximately 53,882 of total population [16]. However, the reported total population might be underestimated. The location of most government offices requires Kei Besar people to travel by ship, leading to a weak registration system [17]. In addition, there are babies born in informal facilities who did not receive birth records or certificates [18]. The Kei Besar District is divided into 37 *ohoi*s, the smallest administrative unit equivalent to a village [19].

#### Socio-demographic and economy

The majority of Kei Besar people are Roman Catholics, with a smaller proportion of Protestant Christians and Muslims [19]. Only 73.22% of the citizens aged 16–18 years old attend school, and most people work in their own fields [19]. The soil is mostly poor and rocky. People practice slash-and-burn agriculture to grow cash crops such as copra and tubers which serve as their staple food, as well as grow vegetables in their yards [11]. Some people also catch fish, collect clams and seaweed, or hunt wild boars in the forest [11].

Most of the people in Kei Besar Island belong to one ethnic group, called the Evav tribe, and speak the same language [11, 20]. Today, the native language of Kei people, *Veveu Evav*, is mostly spoken and understood by people over 60 years old, but used rarely by younger people [20]. The majority of Kei people speak in Ambonese Malay or for the younger generation, Bahasa Indonesia, the national language of Indonesia [11, 20].

#### Health facilities

On this island, there are 11 Community Health Centers (*Puskesmas*) spread across 5 districts; eight of which were served by one primary care physician for each center [21]. There is also a non-governmental organization called doctorSHARE in a village near the district capital that offers primary care and nutrition services [22]. There are 46 integrated health services post (*Posyandu*) in each village of Kei Besar district [23], where the community and health providers support the government programs by providing health services to reduce maternal, infant, and under-five mortality rates [24].

### Study design

This study, conducted in January 2023, follows an ethnographic approach to explore the food beliefs and practices relating to pregnancy among pregnant women and key stakeholders such as healthcare providers and traditional healers in the Kei Besar Island.

### Sample procedure and requirement

Married pregnant women aged 18 and above were chosen purposively from seven *ohois* in Kei Besar Island to be included in the study. Women must be a native of Kei Besar Island and able to articulate the cultural values of her communities. Well-known community health volunteers (*kader kesehatan*, villagers with no formal health training who acted as the local public health center’s aide) and traditional healers (*mama biang*, who help with alternative antenatal and delivery services and knows how to make herbal concoctions) were contacted to be informants through word of mouth or recommendations from the villagers. All participants were females. Recruitment was conducted with the help of the staff of doctorSHARE and local midwives from *Puskesmas*. A poster was used to inform the patients of the clinic about the study. Further explanation and inclusion criteria check was done by the first author and doctorSHARE staff through phone call. Twelve pregnant women and five key informants were included in the study (Table 1).

**Table 1:** Demographic characteristics of study participants

Characteristics	Number	
**Pregnant Women**		
Age group 18 19–25 26–30 31–35 * ≥* 36		1 3 4 2 2
Gravidity Primigravida Multigravida		1 11
Educational Level Primary school High school/ Vocational High School		3 9
*Ohoi* location Coastal Mountain		7 5
Occupation Housewife Teacher Shop owner Subsistence farmer	6 2 1 3	
**Other Community Informants**		
Age group 30–40 41–50 51–60 61–70 *≥* 71	1 1 0 1 2	
Years of Experience < 10 years > 10 years	2 3	
Education Level Primary school Junior high school High school/Vocational school	1 3 1	
*Ohoi* Location Coastal Mountain	3 2	
Occupation Community health volunteer Traditional healer	2 3	

### Data Collection methods and tools

**Fig. 1:**
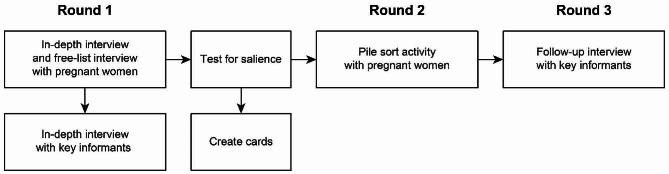
Stages of data collection on Kei Besar Island

The data collection process consists of three rounds, utilizing the principle of emergent design. The data collected in each round informed subsequent rounds, allowing for a deeper understanding of the phenomenon and ensuring that the research design remained responsive to the emerging data and insights (Fig. 1). We developed interview guides for use during the data collection process and have uploaded them as supplementary materials for this article. Please refer to Additional File 1 for the in-depth interview guide for pregnant women, Additional File 2 for the in-depth interview guide for community health volunteers, and Additional File 3 for the in-depth interview guide for traditional healers.

In the first round of the data collection, pregnant women were asked to list the types of food commonly consumed by their community or family members. This was followed by a secondary question about the benefits or adverse effects of the mentioned foods on pregnancy. Then, the women were interviewed with a set of open-ended and close-ended questions to cover topics about their personal experiences with food proscriptions and prescriptions during pregnancy, their efforts to maintain their health during pregnancy, and interpersonal and community influences on their food choices. Interviews were conducted by the first author in Ambonese Malay at the participants’ houses. Interviews were digitally recorded and lasted for approximately 50–90 min. The second author also conducted unstructured observations of the participants’ physical and food environments in their households. The information gleaned from these observations was utilized to provide context for the authors’ understanding of food sourcing, preparation, and consumption within the community. Interviews with the key informants covered similar topics to get a more in-depth explanation and clarity of the findings from the previous interviews.

In the second round, salient items were collected from the answers to the free list questions based on frequency. These items were made into cards that will be used in the pile sort activities. Pile sort activities include free pile sort and structured pile sort with the question “which food is good for pregnancy?”

In the third round, the key informants were interviewed to get more understanding about food classifications or categories in the community. The opportunity was used to clarify new information obtained during the second meetings with the pregnant women.

### Data analysis

The digital recordings of the interviews were transcribed verbatim and translated into English by the first author and coded in Atlas.ti Windows (Version 23, Scientific Software GmbH) to identify categories, patterns, and themes. All food items mentioned in response to the free list questions were entered into Microsoft Excel (Microsoft 365, Microsoft Corporation) to have their frequency counted, leading to salient items identification. Pile sort results were written into a proximity matrix and analyzed using Visual ANTHROPAC (Analytic Technologies, Lexington, KY).

## Results

### Foods proscribed (restricted) during pregnancy on Kei Besar Island

Participants mentioned several food items that should be avoided or minimized during pregnancies (Table 2).

**Table 2:** Food restricted during pregnancy and breastfeeding period

Food Type	Underlying Reasons	Percentage (*n* = 17)
Certain types of fish (*Komo*,* momar*)	Causes itch to the mother, during breastfeeding period causes stomachache and restlessness to the baby	53
Chili and spicy food	Causes eye problems if consumed during pregnancy. Causes the baby to cough if consumed during breastfeeding period. May cause red rashes on the body and make the child naughty as they grow up	47
Soda	Causes miscarriage	41
Pineapples	Causes miscarriage	41
Octopus and squid	Causes red rashes on the baby, increased fetal movement, high blood pressure on the mother, causes difficulty in labor and possible molar pregnancy	35
Papaya leaves	High blood pressure, miscarriage if consumed during early pregnancy, causes baby’s leg to jerk if consumed during breastfeeding period	35
Ice	Makes babies big	30
Clams	Causes stomachache to the mother, causes baby to cough if consumed when breastfeeding, stillbirth	30
Alcohol	Causes miscarriage	24
Rice	Makes babies big, if consumed too much in late pregnancies	24
Meat	High cholesterol and fat may cause health problems to the mother and fetus	18
*Ganemo* (melinjo) leaves	Consumption during breastfeeding will cause cough and restlessness of the babies’ legs at night	18
Red coconuts	Causes increase in body heat which endangers the fetus	18
Crabs	Same reason as octopus and squids	12
*Petatas* (sweet potato) leaves	Causes babies to cry easily and have stomachache	12
*Rebong* (bamboo shoots)	Causes babies to cough and not able to sleep	12
Oily food	Causes babies to cough and dislikes the taste that transfers through breastmilk	12
Sour food	Causes *sakit maag* (gastritis)	12
Avocado	Makes babies big and fat	6
*Kenari* (pili) nuts	Makes babies big and fat	6
Watermelon	Makes babies big	6
Sago	Makes babies big	6
Coffee	Causes babies to have skin discoloration, coffee-colored skin and complexion	6

#### Certain types of fish

Nine out of 17 informants (53%) mentioned that certain kinds of fish are better avoided or minimized during pregnancy. These include fish that have been stored in the freezer, *komo* (black skipjack; *Euthynnus affinis*), and *momar* (scad fish; *Decapterus sp.*) [25, 26], due to the tendency of pregnant women to develop allergies and itchiness after consuming these types of fish. The *mama biang* also talked about how eating *komo* fish during pregnancy may cause the babies to have stomach discomfort.

“*Komo* fish is different for different people, some people develop allergy after eating those, some pregnant women they can also itch from eating those” (Pregnant woman, 31–35 years).

“*Komo*, papaya, papaya leaves, *petatas* leaves, all those will cause discomfort in the stomach, so it’s disease. Later when the child is grown, then they [pregnant women] can eat those, after the child has been weaned from breastmilk.” (*Mama biang*, 61–70 years).

#### Chili or spicy food

Eight participants (47%) reported that chili and spicy food should be restricted during both pregnancy and breastfeeding. Participants believed consuming chili and spicy food during pregnancy could lead to a range of eye problems, such as eye crusts, red eyes, big eyes, and small eyes. Additionally, chili and spicy food can also be passed through the breastmilk and cause the baby to cough.

*“*They said it might affect the child’s eyes. Usually… their eyes will be red, even if they’re still closed.” (Pregnant Woman, 31–35 years).

“If the mother has started breastfeeding… Same… if you eat spicy food, your children will have a lot of *tai mata* (eye crusts)… and they will cough… from the breastmilk…” (*Mama biang*, *≥* 71 years).

#### Soda and alcohol

Seven participants (41%) reported that soda or carbonated drinks should be avoided by pregnant women as they may cause disability or miscarriage. The same beliefs are also held by some participants (23%) about alcohol. Both beverages are considered *keras* (translation: “strong”) and may harm the fetus. The term was used to signify the harm of the beverages toward the fetus.

“I think *sopi* (traditional alcohol from Eastern Indonesia, made from fermented aren/nira palm fruits)… you can’t drink… Usually if you drink *sopi*, it could cause miscarriage to the child as well.” (Pregnant woman, 19–25 years).

“You should not eat… drink… like *xx*, or *yy* (two famous brands of carbonated soft drinks)… some people said they had miscarriage… maybe the drinks are too hard, doc.” (Pregnant woman, 31–35 years).

#### Pineapples

Pineapples were mentioned by some participants as the cause of miscarriage (7 participants, 41%). It is implied that pineapples are believed to compromise the womb and trigger miscarriage or affect the fetus directly.

“What they can’t… for pregnant women, some sort of fruits, like pineapples… pregnant women can’t eat pineapples… because the young ones… they affect… I mean…pineapples, if people are… in their young pregnancy, if you eat pineapples… they can destroy the fetus immediately. Then the mother will have a miscarriage.” (Community health volunteer, 30–40 years).

#### Octopus and squids

Around 35% of participants mentioned that they should not eat octopus and squid, with reasons varying from adversities toward the newborn, difficulty in giving birth, to following elders’ advice. One participant mentioned that eating octopus and squid is restricted due to their tendency of causing high blood pressure, while one stated they might cause the fetus to move a lot inside the womb. A key informant also cited that the elders attributed molar pregnancy (*hamil anggur*) to eating octopus. One of the participants told the interviewer that eating crabs have the same effects.

“They said for octopus, squid, the child will move forward and backward, so when we push them out, they move forward a bit, and then they will go backward again. That makes it difficult to give birth.” (Pregnant woman, 19–25 years).

#### Papaya leaves

An equal proportion of participants (35%) also mentioned papaya leaves unsuitable for consumption during pregnancy and breastfeeding period. Papaya leaves are believed to bring potential risks of miscarriage during early stages of pregnancy, high blood pressure for mothers, and ailments for breastfeeding babies.

“If you eat those [papaya leaves], the children will cry easily… cry a lot… then it will be hard for them to sleep at night… then… from breastmilk… Usually the babies’ legs will be *tacigi-cigi* (jerking), like they’re having seizures.” (*Mama biang*, 61–70 years).

### Food prescribed (recommended) during pregnancy

Participants generally reported that their health providers recommended rice, vegetables, and fish (other than ones mentioned above) as foods that should be consumed during pregnancy. However, some food items are endorsed for more consumption. We present the summary of food prescriptions and the underlying reasons in Table 3 and explain the top findings according to frequency.

**Table 3:** Food recommended during pregnancy and breastfeeding period

Food Type	Reason	Percentage (*n* = 17)
Cassava leaves	Increases breastmilk production, nutritious for the mother and baby, increases blood	59
Papaya	High vitamin content, cleanses the baby upon birth	53
Coconut water	Cleanses baby upon birth, increases and clears amniotic fluid	53
Rice	Important nutrient for developing fetus during early pregnancy. Red rice can increase blood, black sticky rice is used as supplemental food	41
*Kelor* (moringa) leaves	High in vitamin, increases breastmilk production, increases blood	35
Banana	Healthy and nutritious, high in vitamin, helps defecation	35
*Katok*	High in vitamin, increases breastmilk production, increases blood	35
Spinach	High in vitamin, increases breastmilk production, increases blood	35
*Petatas*	Satiating, prevents nausea, does not cause epigastric pain	24
*Petatas* bud	Increases blood	18
Milk	Boosts pregnant mother’s health, the milk for pregnant women prevents nausea	18
*Petatas* leaves	Increases blood, helps with defecation	18
Mango	High in vitamin, decreases nausea, helps with defecation	18
*Keladi* (taro)	Satiating, doesn’t cause nausea and vomiting	18
Mustard	Nutritious, increases breastmilk production	12
Eggs	Gives nutrition and protein	12
Papaya leaves	Increase immunity, lower blood pressure	12
Cucumber	Lowers blood pressure	12
Avocado	Nutritious, increases fetus weight	12
*Ganemo* leaves	High in nutrition, increases breastmilk production	12
Egg whites	Make baby’s skin clear and bright	6
*Gedi* (Aibika)	Nutritious	6
Corn	Satiating	6
Long beans	High in vitamins	6

#### Cassava leaves

*Embal* is often used to describe the flour or food products made from poisonous *kasbi* (cassava) but is also what the locals call the poisonous cassava itself. Around 59% of the participants said cassava leaves should be consumed when pregnant and after giving birth. Most participants said cassava leaves are highly nutritious and help increase blood (*tambah darah*), but most importantly, it increases breastmilk production.

“Well… for pregnant women… just cassava leaves right, we have to eat them to prepare, so that our breastmilk would be increased after we give birth, so it’s recommended…” (Pregnant woman, 19–25 years).

#### Papaya

During pregnancy, papaya is advised for consumption due to its high nutritional value, particularly its vitamin content. Fruit consumption, including papaya, is communicated to pregnant women through their routine check-ups at the local *Posyandu*, which service is provided monthly. Nine participants (53%) mentioned papaya as a prescribed food item. Papaya was also mentioned to have a cleansing property, for the baby when they are born, and for the insides of the womb.

“For advice, I’ll just say, that the papaya makes the baby’s skin clean… because that’s what I hear from the midwives right… with coconut water too… maybe you shouldn’t eat pineapples because I heard that it affects the womb too, right.” (Community health volunteer, 41–50 years).

#### Coconut water

Coconut water was mentioned by 53% of the participants because it is said to clear and increase the volume of amniotic fluid, easing labor. In addition, the cleansing attribute of the food is sought after in the hopes of promoting the delivery of a newborn with clean and unblemished skin.

“I regularly drink fresh young coconut water now… Because having fresh coconut is good for your health… My mother told me that consuming young coconuts makes the child clean during labor. I don’t want the baby to look dirty when my baby is born…like being born covered with too much blood.” (Pregnant woman, 19–25 years).

#### Fish

While *komo* and *momar* fish were reported to cause allergies if consumed in large amounts, fish is recommended by 53% of the participants and recognized as an important source of nutrients. However, fish consumption is dependent on the climate and the resulting price in the market. The condition for fishing in Kei Besar is significantly affected by the monsoon winds, causing higher tides and stronger winds during the western (December, January, February) and eastern monsoon periods (June, July, August) compared to the transition period (March, April, May) [27].

“Red fish (red snapper) are good, nutritious. The meat gives… nutrition for the fetus, like that.” (Pregnant woman, 31–35 years).

“If it’s *batu-batu* fish (fish found around the coral, such as groupers or parrotfish), the ones with many scales, or *bubara* (trevally) fish, the ones that are meaty, they don’t cause problems. But only when you have it, if you don’t have, you can substitute with eggs.” (Pregnant woman, 26–30 years).

### Suggested Dietary Behavior during pregnancy

Dietary behaviors in the community are mainly practiced to prevent adversities for the fetus and ensure safe delivery. There were 11 practices that emerged during the data collection process. We will explain the most frequently found.

#### Eating for two

Six participants talked about increasing their food intake to feed the fetus. Eating two portions of a meal is believed to be necessary to ensure the fetus grows accordingly and is even endorsed by health providers such as midwives and community health volunteers in *Posyandu*.

“I always tell them to eat more than one plate because they eat for more than one person, they have to eat many vegetables, many fish, from the 0 month of their pregnancy, they have to eat nutritious food so the child will not be stunted, until two years old, the child has to eat nutritious food.” (Community health volunteer, *≥* 71 years).

Other than the importance of the fetus development, it is also believed that a pregnant woman should eat for two to prepare her to produce more breastmilk.

“They have to eat a lot, more than usual. So that they can have a lot of breastmilk. If they eat less… they will not have enough breastmilk, right, so they have to eat a lot to be able to breastfeed their children.” (*Mama biang*, 61–70 years).

#### Using smaller plates

Despite generally agreeing with the idea of eating more than usual during pregnancy, 30% of participants also stated the necessity of eating with smaller containers or plates. Eating with bigger plates is believed to cause the placenta to be large and cause difficulty when delivering the baby later.

“We should not use big plates… we have to use smaller containers… They [older family members] said… they fear that later *de pu kaka* (literally translated as “the older sibling”, how the locals refer to placenta) will be bigger, so we have to eat using small plates. (Pregnant woman, 31–35 years)

#### Reducing food and rice intake (Eating Down) in late pregnancy

Furthermore, at some point, pregnant women should stop eating for two. 24% of participants mentioned that when pregnancy enters the third trimester, pregnant women should reduce food and rice intake to prevent giving birth to big babies. Women learned about this practice from the health providers and their community. Interestingly, other food items such as tubers are believed to not cause weight increase in the fetus as much as rice.

“She [The midwife] said we have to reduce the rice intake… because too much rice, the baby will be big, it would be hard to give birth… starting from 8 months of pregnancy or so, reduce your rice consumption… if you have the *makanan kebun* (food from the field), then eat *makanan kebun*.” (Pregnant woman, *≥* 36 years).

#### Portion rationing

The practice of different portion rationing in families was also mentioned by 24% of the participants. It is recognized by participants as customary in the Kei Besar community to put the elderly and the husbands first in terms of food portion rationing, although it may not be widely practiced. There was no specific mention of the children’s portion or when they should be served food.

“Then my husband… we separate his rice, we separate his vegetables… like that. So, we put rice on one plate, and vegetables in a bowl (*laughs*), that’s just how Kei people are, if we prepare food, we can’t just serve it as we please for our husband. For my husband, or our parents… usually we take separately for them, rice, vegetables, fish… us women, it doesn’t matter, as long as we eat. If… us here, that’s just custom, though, usually people say the head of the fish is important for the husbands (*laughs*), so there has to be a special fish for husbands… like that.” (Pregnant woman, 26–30 years).

#### Other practices

Other practices were reported by two participants or less. These include some common practices to avoid ailments, such as washing vegetables before cooking, covering food served on the table, and eating at regular hours. Other practices include: restrictions on eating with cracked plates or drinking with cracked cups, believed to cause the baby to be born with incomplete limbs; serving warm and not hot food, because it would harm the fetus in the womb; and eating until the plate is completely empty so the baby will be clean upon birth.

### Foods consumed most commonly by pregnant women

The in-depth interviews with pregnant women included a free list question that asked for the types of food commonly consumed in their *ohoi*s. The responses were compiled and analyzed to identify the most frequently mentioned food items, which were deemed as salient (Table 4).

**Table 4:** Free list results of Foods commonly consumed on Kei Besar Island (*n* = 12)

Food Item	Percentage
Rice	92
Banana	83
*Petatas* (sweet potato)	83
*Keladi* (taro)	75
*Komo* fish (black skipjack)	75
Papaya leaves	59
*Kasbi* (cassava)	59
Kangkong	59
*Embal* (hard, dry food made of *kasbi* flour)	50
*Kasbi* leaves	50
Spinach	50
Papaya	50
*Momar* fish (scad fish)	50

**Fig. 2:**
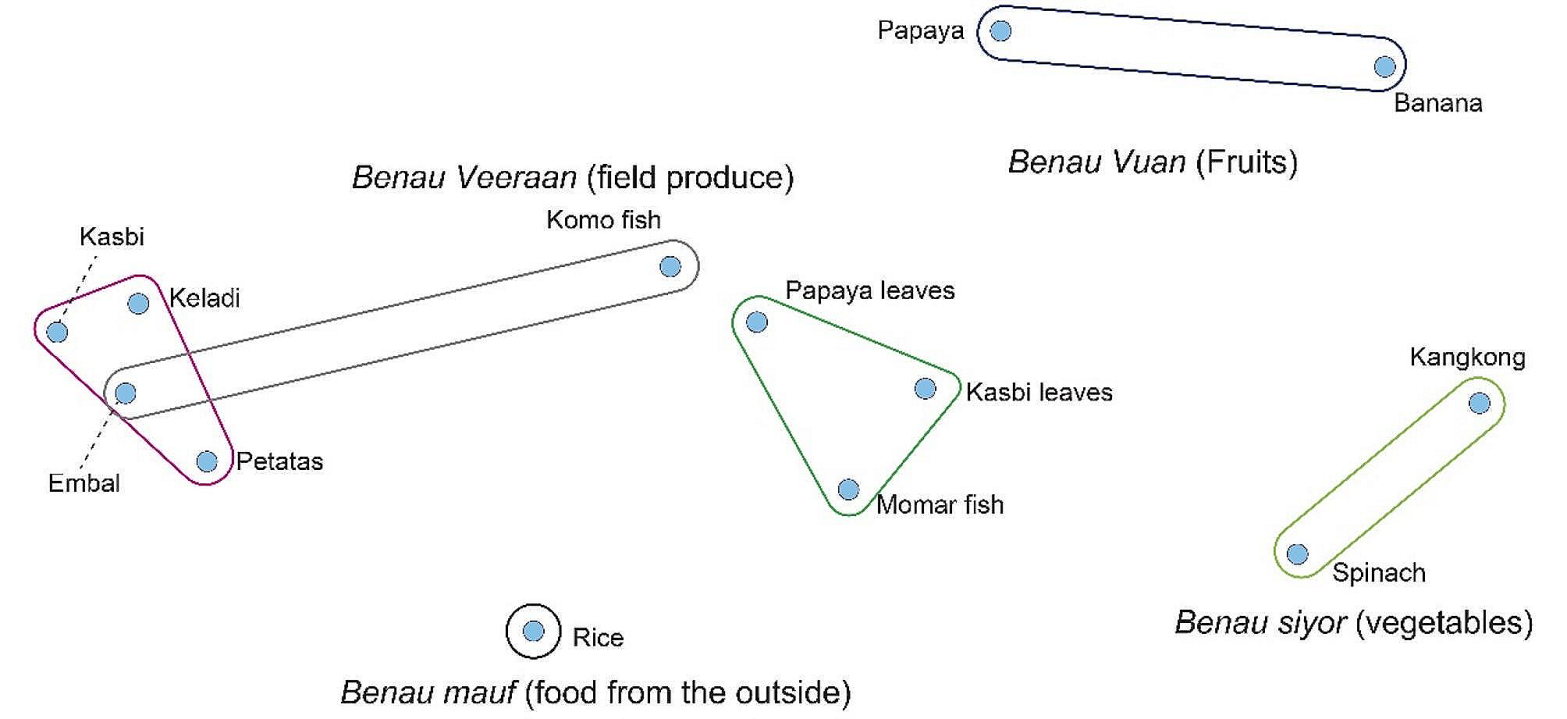
Food groupings of Kei Besar women, free pile sorts

To facilitate further analysis, salient food items were translated into cards containing both the name and picture of the items to be used in the pile sort activity. The pregnant women were asked to sort the cards based on what makes sense for them (free pile sort) and based on the question, “what foods are good for pregnancy?” Pile sort results were analyzed using Visual ANTHROPAC and the resulting multi-dimensional scaling is shown in Fig. 2:

The clusters showed the kinds of food that are considered conceptually similar to each other. *Benau vuut* (fish) are grouped with the food that they go best with. The tubers and their parts such as leaves are classified as field produce, and with fruits and vegetables grown in the yards, form the group called “*Benau Evav*,” translated as “food of Kei people.” Rice is not categorized as food from the field or food of Kei people because it was introduced first by the government and not a native plant of Kei Islands. However, it is consumed by almost all Kei people now. This figure illustrates the food eaten by the pregnant women and the diet commonly found among Kei Besar people.

The structural pile sort question yielded two or three piles from each woman, with them grouping food as “good for pregnancy,” “not too good or doesn’t matter,” and “not good for pregnancy.” The second option indicates the food item has no specific effect on pregnancy, except if it is consumed in a large amount, which may pose health risks. We ranked these piles with weights: two for “good” food, one for “not too good” food, and zero for “not good” food, as seen in Table 5.

**Table 5:** Food benefits for pregnancy ranking (*N* = 11)

Food item	Good	Not so good/doesn’t matter	Not good	Total
Banana	10	1	0	21
Rice	10	0	1	20
Spinach	9	2	0	20
Papaya	8	3	0	19
*Komo*	7	4	0	18
Kangkong	6	4	1	17
*Momar*	8	1	0	17
*Petatas*	5	6	0	16
Embal	4	6	1	15
*Kasbi* leaves	5	5	0	15
*Keladi*	4	7	0	15
*Kasbi*	4	6	0	14
Papaya leaves	2	6	3	10

Five participants mentioned the reason they placed food items under the “good” category is because they heard about the food items’ nutritional benefits from the community health volunteers or *Posyandu* nutritionist, such as rice helps with development during the early years, or fish makes children smart. This implies that pregnant women will consider a food item “good for pregnancy” if it promotes fetal health. Three other participants mentioned that food in the “not so good” category (*embal*, kangkong, *keladi*) may cause an increase in stomach acid and lead to gastritis if consumed too much. The key informants then elaborated that there are food items known as “*benau vusin*,” which translates to “hard food” and “*benau mafun*,” which translates as “soft food.” The hard food causes *saki ulu hati* (epigastric pain) if consumed too much, while the soft food does not. There is also a community belief to not eat too many leafy vegetables since it would lead to an increase in uric acid and knee pains.

Many of the foods that are prohibited in the previous section are not listed by the women in the free list session or listed low down the list and did not make it to the pile sort activity. This indicates that those food items were not consumed daily by the people of Kei Besar Island.

## Discussion

As per the authors’ research, this is the first study to explore the food beliefs and practices regarding pregnancy in Maluku province, Indonesia. The use of different informants and incorporation of different methods gives different viewpoints that resulted in a more holistic picture of the culture and practices surrounding the dietary behavior among pregnant women.

Food choices among participants are influenced by important referents such as healthcare providers in *Posyandu*, mothers or mothers-in-law, traditional healers, and the advice from community elders that are passed intergenerationally. Considerations of food choices also take into account the seasonal availability of food, such as the monsoon winds to catch fish, and thus the price of the food item in the market.

During pregnancy, foodstuffs were mainly restricted to ensure a safe and easy delivery, including to prevent miscarriage and to have a healthy baby. This is supported by a practice of eating down in the third trimester of pregnancy, which is reported in other parts of Indonesia [28, 29] and internationally [30, 31]. Avoidance of food items for symbolic reasons, such as squid, octopus, and crabs that make the baby move backward and forward when giving birth has been reported in other studies [32, 33]. In Kei Besar Island, the authors’ conversations with other community members brought about the reason to desire a smaller baby, which mainly relates to the fear of having a Caesarean Section, believed to cause immense pain and will interfere with the mothers’ ability to work in the field.

The same practices of proscribing some food items during breastfeeding period and prescribing the ones believed to increase breastmilk production is also seen in other parts of Indonesia [29, 34]. We did not find food prescriptions and proscriptions based on the “hot-cold” system that exists in some parts of Indonesia or other countries [29, 30, 34–36].

Other than to increase breastmilk, food that is believed to increase blood, thought as aiding in delivery, are also recommended. “Lack of blood” (*kurang darah*) in pregnancy is believed to cause health issues such as weakness or dizziness, while “dirty blood” should be expelled after delivery because it could go to the head and cause an infirmity called *darah putih*, where the women will have terrible headache and faint, even to the point of losing sanity or death. To increase blood, the pregnant women routinely take the *tablet tambah darah* (translated literally as blood-increasing tablets) from *Posyandu*, which contains iron and folic acid. To prevent *darah putih*, they take traditional medicine to clean the wombs. The importance of food that could help increase blood to prepare for labor is found in some studies [30, 31, 37], but there is also a study that document avoidance of food that cause increase of blood, which is believed to induce hemorrhage during delivery [28].

The appearance of a baby upon birth is an important factor in deciding to consume a food item during pregnancy. Babies are desired to have a clean appearance and clear or bright skin, while a dark complexion is undesirable, similar to the beliefs that have been documented elsewhere [31, 35, 37].

Despite the Kei Besar people belonging to one ethnical group, the idea of food items that should be strictly avoided or okay to consume in moderate amount may differ from person to person due to differences in individual experiences and the information they have received and applied in their daily lives [38]. This cultural variation can also be a topic for future studies and should be noted when designing interventions. Nevertheless, pregnant women from different educational statuses and types of *ohoi* of residence (coastal or mountain) shared certain food proscriptions or prescriptions, indicating that these two variables did not affect their beliefs.

The recommendations from community health volunteers, midwives, or nutritionists at *Posyandu* seemed to be especially influential in pregnant women’s food choice. This is noted from some participants categorizing fish (even if the ones included in the pile sort exercise were believed to be better minimized) as “good for pregnancy” in the pile sort activity since it was recommended by the health workers at *Posyandu*. As previously mentioned, pregnant women prioritize a safe delivery and a healthy baby. Hence, they heed advice from credible sources such as community health volunteers and health professionals regarding foods that may offer health benefits to the fetus and future child, such as aiding intelligence or supporting intrauterine development. Consequently, some pregnant women may consume foods that they were advised to restrict, over considerations that the health benefits may outweigh the risks, if not consumed excessively. This gives a room for a development of intervention that involves capacity building for health workers to provide culturally appropriate nutritional education, and to make sure nutritional contents are met by local food ingredients, to complement the food ingredients recommended in the KIA (*Kesehatan Ibu dan Anak*; Maternal and Child Health) book circulated by the Ministry of Health. Traditional healers and family members are potential targets of health promotion efforts to encourage a healthy diet during pregnancy.

This study has several limitations. First, while we were able to uncover the influence of these beliefs on the pregnant women’s food choices, we did not examine the women’s actual diet by obtaining dietary recalls, prohibiting us from inferring that the food beliefs and practices actually resulted in a poor diet that may influence nutritional status. Second, the small number of pregnant women in the *ohoi*s we visited resulted in less various informants in terms of gestational age and parity, which are related to the individual experiences of the informants. Other types of informants such as mothers or community elders could be approached to obtain more insights on these food proscriptions and prescriptions during pregnancy. Lastly, some participants recognized the first author (interviewer) as a physician, which may lead them to give responses that are considered “right” for the author. These limitations should be considered while interpreting this study’s findings. Future studies should depart from the fact that these beliefs exist and combine analysis using tools such as dietary recalls and food diaries on more pregnant women to determine the effects of these beliefs on the nutrient intake and nutritional status of the pregnant women. Studies involving pregnant women with CED and varying nutritional statuses from the same community could also be conducted to assess which good practices are beneficial during pregnancy, taking into account existing beliefs found in this research. Furthermore, studies to explore the knowledge of community health volunteers and health professionals from *Posyandu* or *Puskesmas* will be necessary to inform the development of interventions aimed at improving knowledge or building capacities to provide culturally appropriate health and nutritional education.

## Conclusions

The results of this study indicate that food beliefs are present in Kei Besar, as in other parts of Eastern Indonesia. Recommendations for certain foods such as moringa leaves, as well as prohibitions on others, such as alcohol, can be beneficial to health and should be emphasized during ANC (Antenatal Care) sessions at *Posyandu*. However, proscriptions of some food items (such as seafood) may decrease the quality of nutritional intake and advises against them should be revisited. Pregnancy dietary recommendations should include substitute foods for those that have long-standing restrictions. The influence of community health volunteers and providers in *Posyandu* highlights the need for training and intervention to update their knowledge of nutritious food and to ensure consistent information is provided to benefit pregnant women.

### Electronic supplementary material

Below is the link to the electronic supplementary material.

Additional file 1.pdf. Title of the data : Interview Guide for Pregnant Women.
Description of the data: This file consisted of two interview guides for pregnant women, the first one is for the first interview (first round of data collection) and the second guide is for the pile sort activity (second round of data collection).

Additional file 2.pdf. Title of the data : Interview Guide for Community Health Volunteers.
Description of the data: This file consisted of two interview guides for community health volunteers, the first one is for the first interview (first round of data collection) and the second guide is for the follow-up interview (third round of data collection).

Additional file 3.pdf. Title of the data : Interview Guide for Traditional Healers (“Mama Biang”).
Description of the data: This file consisted of two interview guides for traditional healers (*mama biang*), the first one is for the first interview (first round of data collection) and the second guide is for the follow-up interview (third round of data collection).

## Data Availability

The data analysed during the current study are not publicly available due to the potential of containing participants’ personal or family information, but are available from the corresponding author on reasonable request.

## Declarations

### Authors’ information

JGM is an Indonesian primary care physician who trained in North Sulawesi and practiced in Kei Besar Island for a year, gaining conversational proficiency in Ambonese Malay. PM, a physician who also studied at Hopkins accompanied JGM to Kei Besar Island, providing support by recording pile sort groupings and participating in peer debriefings. Both authors reported the field conditions to JG, an experienced medical anthropologist who served as a content expert and provided guidance remotely during the fieldwork. ON and AS are both experienced public health physicians and researchers from doctorSHARE who provided support on the field and during the manuscript writing process. IP is a research associate from Hopkins who provided substantial input and revision on the manuscript.

## Abbreviations

CED: Chronic Energy Deficiencies
DALYs: Disability-adjusted Life Years
KIA: Kesehatan Ibu dan Anak; Maternal and Child Health
ANC: Antenatal Care
