# Supplementary material for: Formative research to understand food beliefs and practices relating to pregnancy on Kei Besar Island, Eastern Indonesia
Source: BMC Nutr. 2024 Jul 11;10:97. doi: 10.1186/s40795-024-00905-2 (PMC11238455; doi:10.1186/s40795-024-00905-2)
Supplement: Supplementary file 1 — Additional file 1.pdf. Title of the data : Interview Guide for Pregnant Women. Description of the data: This file consisted of two interview guides for pregnant women, the first one is for the first interview (first round of data collection) and the second guide is for the pile sort activity (second round of data collection). [file 40795_2024_905_MOESM1_ESM.pdf]

Interview Guides

Formative research to understand food beliefs and practices relating to pregnancy on Kei Besar Island, Eastern Indonesia

<u>Interview Guide for Pregnant Women</u>

<table>
<tr><td colspan="2">Village:</td><td colspan="2">Geography:
• Rural, coastal zone
• Rural, non-coastal zone</td></tr>
<tr><td>Interview conducted by:</td><td>Date:
_ _ / _ _ / _ _ _ _
DD / MM/ YYYY</td><td>Pregnancy Type:
(Primigravida or Multigravida)</td><td>Participant Code:</td></tr>
<tr><td>Start Time:
HH:MM</td><td>End time:
HH:MM</td><td colspan="2">Audio file name:</td></tr>
<tr><td colspan="4">Introduction:
Thank you for agreeing to talk with me. Please give us the name you would like to be called— please use a nickname or another name other than your real name just for today.

Remember that everything shared here today is confidential. I will be recording our discussion, so I do not forget anything you say. Today I will ask you to share your thoughts about how women should eat and should not eat, what women actually eat, and what influences their diet when pregnant and breastfeeding. I want to know your thoughts about important foods to eat or not to eat during these times, the challenges in getting the right foods, and who makes decisions about the foods and drinks women consume during pregnancy. I also want to know about any advice about eating or nutrition you receive from all different sources, including traditional healers, local health centers, or other people in this community. So, let’s start by getting to know one another.

Ice breaker:
Let’s start off by talking about what you like about living here in Kei Besar Island.</td></tr>
<tr><td colspan="4">Interview Questions

Beliefs about Food and Illnesses:
Free list question to explore local food classification systems
Free list question: What are all the foods that people here in (village name) eat?

Personal Experiences and Social Norms Regarding Pregnancy
1. Tell me about how women in your village should eat during pregnancy and share what you eat now/ate during your pregnancy.
2. What did you hear about how you should eat during pregnancy? How did you hear about this?
a. Probe: Why do you think women should eat or not eat (insert type of food mentioned)?
3. Who told you about how to eat during pregnancy? (e.g., other women, doctors, family members, traditional healers)
4. What are the medications or supplements you took during pregnancy? Who told you to take those medications or supplements? Why do you take them?</td></tr>
</table>

5. (Multigravida) Tell me the story of your pregnancy with your previous child, what happened during your pregnancy?<br>6. (Primigravida) Tell me the story of your current pregnancy.<br>7. Who did you visit for care during your pregnancy and delivery? Why do you visit them? (i.e., Western doctors, community health workers, traditional healers)<br>8. What support do you receive from your family during pregnancy? How do they affect what you eat?

Interview for Pregnant Women – Pile Sort Activity

**Village:**<br>•		**Geography:**<br>• Rural, coastal zone<br>• Rural, non-coastal zone	
**Interview conducted by:**	**Date:**<br>_ _ / _ _ / _ _ _ _<br>DD / MM/ YYYY	**Pregnancy Type:**<br>(Uniparous or multiparous)	**Participant Code:**
**Start Time:**<br>HH:MM	**End time:**<br>HH:MM	**Audio file name:**	
**Opening**<br>Hello, thank you for your time for this second meeting. Today we will do an exercise to learn more about foods that are important during pregnancy. I have cards here, they contain the food items that you and the other pregnant women mentioned during the first meeting. I would like to ask you to group the cards together. Take your time to read the food items written on the card and look at the pictures.			
**Interview questions**<br>1. Pile sort to explore local food classification systems<br>Pile sort question: Please group these foods into whatever groups make sense to you. Why did you group these in this pile? Those in that pile?<br>2. Pile sort to explore perceived benefits of foods for pregnancy<br>Pile sort question: Please group these foods into which are good for pregnancy. Why did you group these in this pile? Those in that pile?			
