# Supplementary material for: Formative research to understand food beliefs and practices relating to pregnancy on Kei Besar Island, Eastern Indonesia
Source: BMC Nutr. 2024 Jul 11;10:97. doi: 10.1186/s40795-024-00905-2 (PMC11238455; doi:10.1186/s40795-024-00905-2)
Supplement: Supplementary file 2 — Additional file 2.pdf. Title of the data : Interview Guide for Community Health Volunteers. Description of the data: This file consisted of two interview guides for community health volunteers, the first one is for the first interview (first round of data collection) and the second guide is for the follow-up interview (third round of data collection). [file 40795_2024_905_MOESM2_ESM.pdf]

Interview Guides

Formative research to understand food beliefs and practices relating to pregnancy on Kei Besar Island, Eastern Indonesia

Interview Guide for Community Health Volunteers

<table>
<tr><td colspan="2">Location of Health Center:</td><td colspan="2">Geography:
• Rural, coastal zone
• Rural, non-coastal zone</td></tr>
<tr><td>Interview conducted by:</td><td>Date:
_ _ / _ _ / _ _ _ _
DD / MM/ YYYY</td><td>Years of Experience:</td><td>Participant Code:</td></tr>
<tr><td>Start Time:
HH:MM</td><td>End time:
HH:MM</td><td colspan="2">Audio file name:</td></tr>
</table>

Introduction:

Thank you for agreeing to talk with me. Please give us the name you would like to be called— please use a nickname or another name other than your real name just for today.

Remember that everything shared here today is confidential. I will be recording our discussion, so I do not forget anything you say. Today I will ask you to share your thoughts about what women eat and their diet when pregnant. I want to know your thoughts about important foods to eat or not to eat during these times, challenges in getting the right foods and who makes decisions about the foods and drinks women consume during pregnancy. I also want to know about any advice about eating or nutrition you give pregnant women or women who are breastfeeding. So, let's start by getting to know one another.

Ice breaker:

Let's start off by talking about what you like about living here in Kei Besar Island.

Interview Questions

Personal Experiences and Social Norms Regarding Pregnancy

1. How long have you served as a cadre/community health volunteer here in the community?
2. Tell me the story of a pregnant woman whom you recently met.
3. Tell me about how women in your village should eat during pregnancy.
4. What did you hear about how women should eat during pregnancy? How did you hear about this?
    a. Probe: Why do you think women should eat or not eat (insert type of food mentioned)?
5. When you meet a pregnant woman, what do you talk to her about?
6. Why did you advise women to take certain medications or supplements during pregnancy? What are these medications or supplements?

Interview Guide for Community Health Volunteers

<table>
<tr><td colspan="2">Location of Health Center:</td><td colspan="2">Geography:
• Rural, coastal zone
• Rural, non-coastal zone</td></tr>
<tr><td>Interview conducted by:</td><td>Date:
_ _ / _ _ / _ _ _ _
DD / MM/ YYYY</td><td>Years of Experience:</td><td>Participant Code:</td></tr>
<tr><td>Start Time:
HH:MM</td><td>End time:
HH:MM</td><td colspan="2">Audio file name:</td></tr>
<tr><td colspan="4">Opening:
Hello, thank you for sparing some time to let me visit you the second time. This time I want to discuss about the findings from the interviews with the pregnant women. Please explain to me about the foods that are the most important, most common, for the pregnant women.</td></tr>
<tr><td colspan="4">Interview Question
1. During my interview with the pregnant women, they mentioned that these are the foods consumed by pregnant women. Which ones do you think are the most important to be eaten during pregnancy? Which is the most commonly eaten? Which ones should not be eaten?</td></tr>
</table>
